# Neuromuscular Dysfunction and Charcot-Marie-Tooth Disease Reversal in Mfn2 T105M Knock-In Rats

**DOI:** 10.3390/ijms27167376

**Published:** 2026-08-18

**Authors:** Jochen Weigele, Antonietta Franco, Gerald W. Dorn

**Affiliations:** 1 Department of Regenerative Medicine and Cell Biology, Medical University of South Carolina, 171 Ashley Ave, Charleston, SC 29425, USA; weigelej@musc.edu; 2 Center for Pharmacogenomics, Department of Internal Medicine, Washington University in St. Louis, Saint Louis, MO 63110, USA; antonietta.franco@hunimed.eu

**Keywords:** mitochondria, neuropathy, myopathy, mitofusin activator, magnetic resonance imaging, electromyography

## Abstract

Charcot-Marie-Tooth (CMT) disease type 2A is a rare heritable disorder caused by pathogenic variants of mitofusin (MFN) 2 that suppress mitochondrial fusion and motility in peripheral nerves, culminating in denervation myoatrophy. The rarity of this condition and the limited choice of animal models preclude pre-clinical evaluation of many tests that could be translated to human trials. Here, we introduced the CMT2A pathogenic variant MFN2 T105M into the rat genome for phenotype characterization and evaluation of disease response to a third-generation mitofusin activator, 8015-P2. CMT2A rats exhibited peripheral motor and sensory neuron dysfunction. Functional, histological, neuroelectrophysiological and magnetic resonance imaging testing readily distinguished between wild-type (WT) and mutant rats via axonopathy and myoatrophy. Compound 8015-P2 reversed CMT2A-linked neuromuscular degeneration in a dose- and time-dependent manner; at 10 mg/kg/d, normalization occurred at 4 weeks. The minimal effective 8015-P2 dose was 2 mg/kg/day. Rapidity of phenotype reversal and primary muscle abnormalities are consistent with extra-neuronal effects of the causal MFN2 DNA variant. These data demonstrate unprecedented utility of the Mfn2 T105M rat as a model of CMT2A, expand the menu of clinically applicable tests that may have use in future human trials, and establish a strong foundation for exploration of extra-neuronal consequences of pathogenic mitofusin variants in non-mouse models.

## 1. Introduction

Charcot-Marie-Tooth (CMT) disease is the clinical moniker for a family of genetically and phenotypically heterogeneous peripheral neuropathies caused by damage to neuronal axons or their Schwann cells [1,2]. Postulated to be a spinal cord disorder in 1886 by the French physicians Jean-Martin Charcot and Pierre Marie [3], the underlying pathology was more correctly localized to peripheral nerves by the English physician Howard Tooth [4]. CMTs are broadly characterized by progressive myoatrophy of distal extremities with preservation of core body and facial musculature; sensory defects are variable. In the modern era, neuroelectrophysiological and genetic testing of CMT patients revealed differences that have been categorized into multiple CMT subtypes [5,6,7,8,9]. Our interest is in autosomal dominant CMT type 2A (CMT2A) that affects neuronal axons, typically manifests early in childhood with stabilization in adulthood, and in most instances is caused by loss of function mutations of the mitofusin (MFN) 2 gene that impair mitochondrial fusion and motility [1,9,10,11]. There are currently no clinically approved disease-modifying therapies for CMT2A, although mitofusin activation and HDAC6 inhibition have shown promise in experimental mouse models [12,13,14,15].

CMT2A mice created using neuron-specific transgenic expression of MFN2 mutants [13,16,17] have limited clinical translatability because mutant MFN2 is only expressed in motor neurons, limiting investigations of sensory and extra-neuronal phenotypes. We recently reported an Mfn2 T105M knock-in mouse [15] having a robust CMT2A phenotype in which systemic expression and normal regulation of the causative *MFN2* variant recapitulate the human condition. However, identification and evaluation of clinically relevant disease markers in mice is limited by their small size, and in vivo testing is constrained to procedures that have been suitably miniaturized.

Here, our goals were to 1. develop a non-mouse preclinical model of CMT2A that genetically and phenotypically recapitulates the human condition; 2. test the efficacy of a candidate therapeutic in the model; and 3. utilize the model and candidate therapy in combination to evaluate disease metrics that might serve as clinical endpoints. Like mice, rats have an *Mfn2* gene that is highly homologous to human *MFN2*, but larger rat size permits a wider array of in vivo testing. Additionally, rats may be more faithful models of neurodegenerative disease than mice [18]. We engineered into the rat genome a human *MFN2* mutation encoding the pathogenic T105M variant that provokes early-onset aggressive CMT2A in human carriers [19,20] and a robust sensory-motor neuropathy in knock-in mice [15]. We report that Mfn2 T105M knock-in rats develop a strong CMT2A-like phenotype that is reversed by daily oral administration of a recently described highly potent mitofusin activator, compound 8015-P2 [15], and can be monitored using clinically applicable techniques such as magnetic resonance imaging (MRI) of muscle volume and electromyography/nerve conduction velocity studies (EMG/NCV) of integrated neuromuscular electrophysiology.

## 2. Results

### 2.1. Creation and Functional Characterization of Mfn2 T105M Knock-In Rats

**Figure 1:**
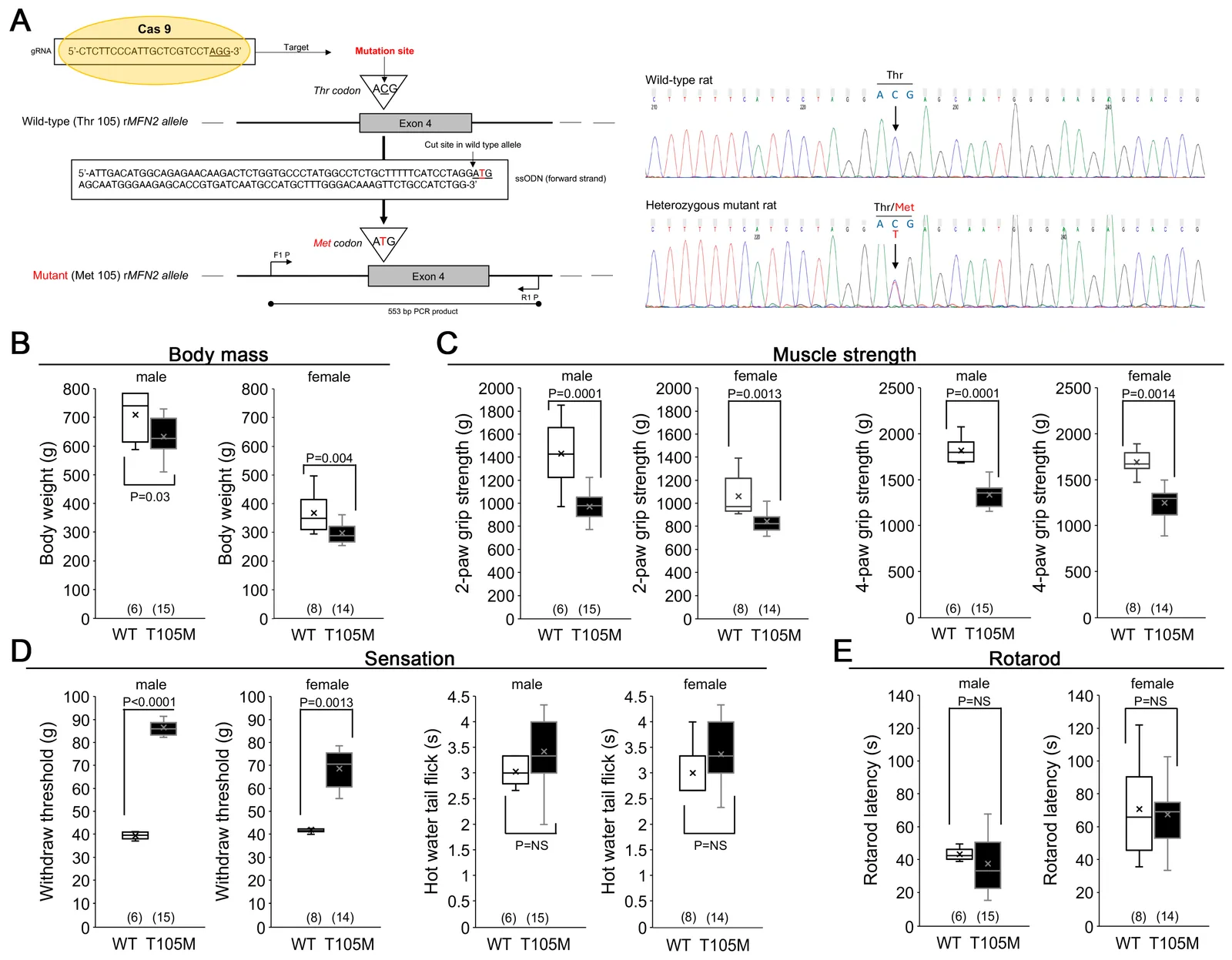
Creation and functional characterization of Mfn2 T105M mutant rats. (**A**) Schematic depiction (**left**) and sequence validation (**right**) of Mfn2 T105M CRISPR/Cas9 rat knock-in. (**B**–**E**) Phenotypic studies of male and female T105M rats (black boxes) compared to wild-type (WT) littermate controls. (**B**) Body mass. (**C**) Front paw (**left**) and 4-paw (**right**) grip strength. (**D**) Von Frey filament testing of front paw tactile sensitivity (**left**) and thermal nociceptive testing measuring tail withdrawal from hot water (**right**). (**E**) Rotarod testing; latency is the time to falling/jumping off.

We engineered the CMT2A-linked T105M variant into the rat *Mfn2* gene using CRISPR/Cas9 (Figure 1A). Heterozygous Mfn2 T105M rats were viable, whereas no homozygous rats were obtained from over 250 pups derived from interbreeding heterozygous Mfn2 T105M (designated CMT2A) rats.

Here and elsewhere, bars encompass the 1st–3rd interquartile ranges, the internal horizontal line is the median value, the internal X is the mean value, and whiskers show the range of values. Animal numbers are shown below boxes above the horizontal axis; *p* values vs. WT are results of an unpaired 2-tailed *t*-test; and NS = non-significant (*p* > 0.05).

Motor and sensory phenotypes were evaluated in 50-week-old rats and their wild-type littermates. Rats exhibit sexual size dimorphism [21], and our male wild-type (WT) and Mfn2 T105M rats were approximately twice the size of their female littermates (Figure 1B). Because size and strength are correlated, neuromuscular function was analyzed separately in the two sexes. Compared to sex-matched WT littermates, male and female CMT2A rats had reduced motor function measured as front (2-paw) and front and back (4-paw) grip strength (Figure 1C); the proportional strength reduction in male and female CMT2A rats was similar. Tactile sensitivity measured at the front paw was reduced in CMT2A rats, whereas tail thermal sensitivity was unchanged (Figure 1D).

In CMT2A mouse models, the inability to stay on a rotating cylinder during progressive acceleration (Rotarod test) can be a sensitive indicator of integrated neuromuscular dysfunction [13,14,15]. Here, both WT and CMT2A rats electively dismounted shortly after initiation of Rotarod testing (Figure 1E), likely reflecting a species-specific difference in behavior [22].

### 2.2. CMT2A Rat Phenotyping Using Clinical Tests

**Figure 2:**
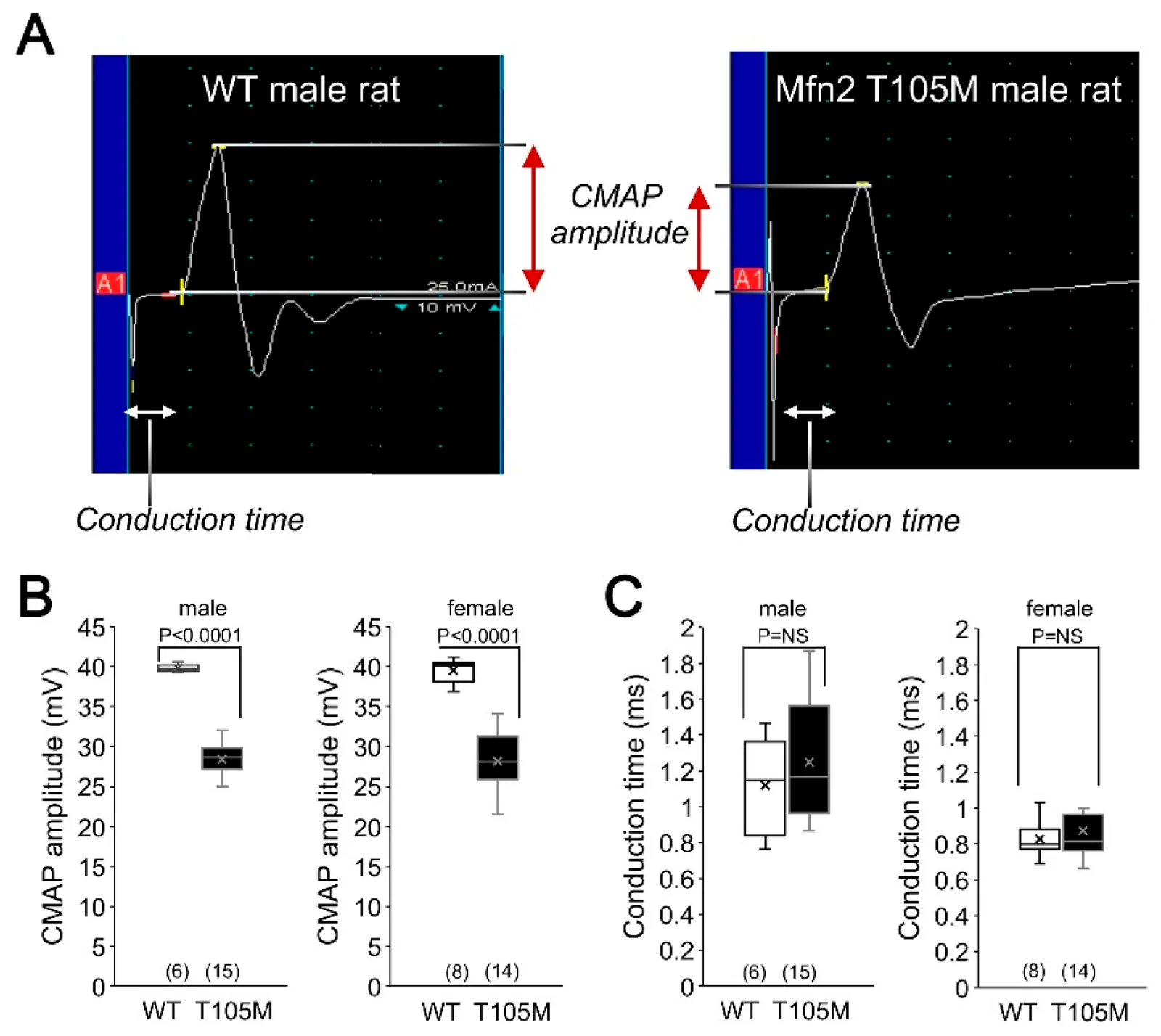
Hindlimb EMG/NCV testing in CMT2A rats. (**A**) Representative 50 wk old male WT (**left**) and T105M (**right**) hindlimb neuroelectrophysiology tracings (sciatic nerve to gastrocnemius/plantaris muscles) depicting compound muscle action potential (CMAP) amplitude and conduction time interval. (**B**) Group data for CMAP amplitude analyzed for males (**left**) and females (**right**). (**C**) Group data for conduction time analyzed for males (**left**) and females (**right**). *p* values vs. WT are the result of an unpaired 2-tailed *t*-test; NS = non-significant (*p* > 0.05).

EMG/NCV testing is a standard clinical test in which a motor nerve is stimulated and the resulting depolarization/repolarization currents in the innervated muscle are measured. In CMT2A, EMG/NCV shows reduced compound muscle action potential (CMAP) amplitude (reflecting loss of neurons and/or muscle mass) and normal nerve conduction velocity (indicating retention of neuronal myelin) [8]. As in the human condition, EMG/NCV testing of hindlimb sciatic nerve-gastrocnemius/tibialis muscles in CMT2A both in male and female rats revealed abnormally low CMAP amplitudes (Figure 2A,B) with normal nerve conduction times (Figure 2A,C).

**Figure 3:**
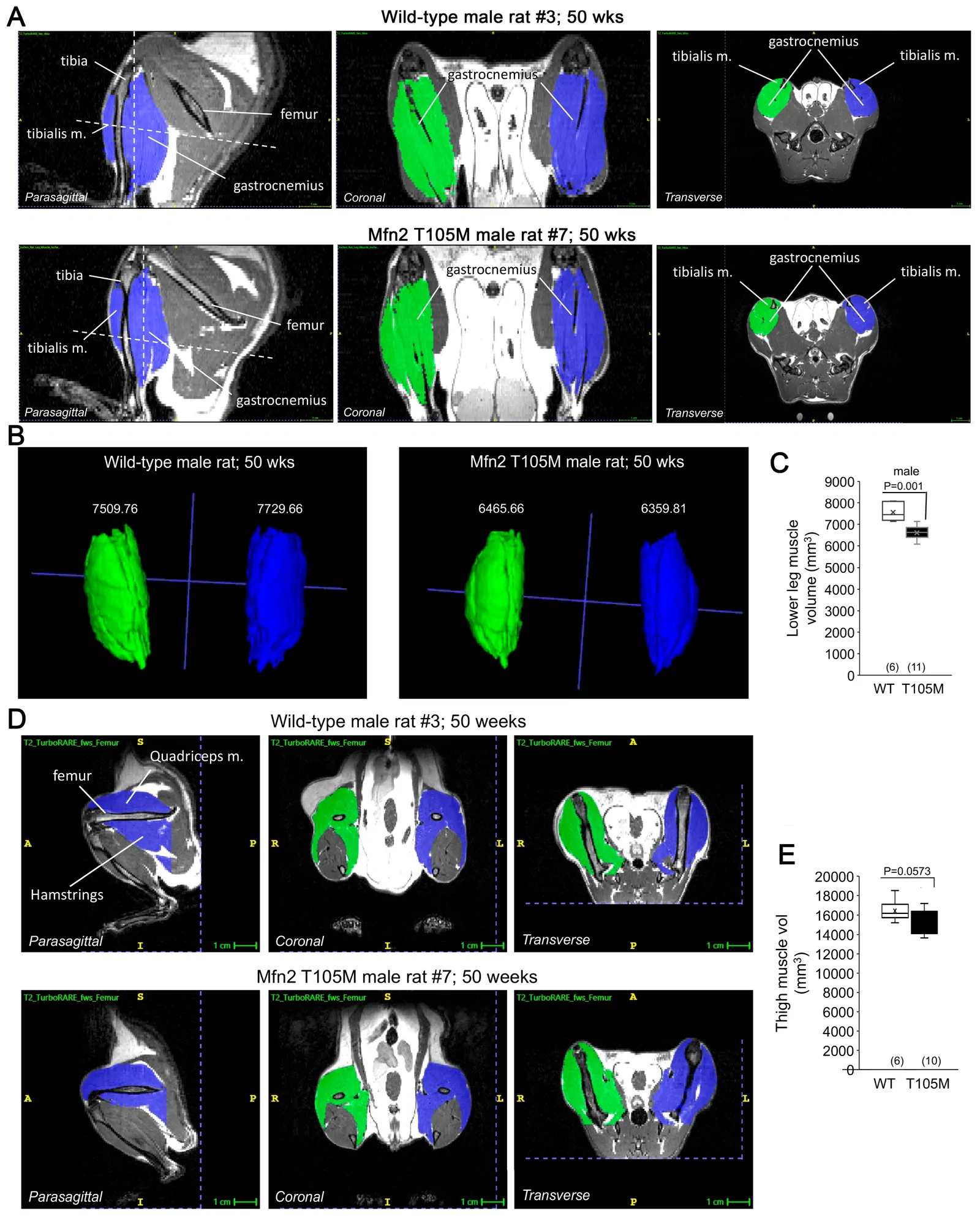
MRI measurements of hindlimb muscle volume in male CMT2A rats. (**A**–**C**); distal hindlimb (calf; gastrocnemius/plantaris/tibialis muscles); (**D**,**E**); proximal hindlimb (thigh; quadriceps and hamstring muscles). (**A**,**D**). Representative images of WT (**top panels**) and Mfn2 T105M KI (**bottom panels**) rat hindlimb in three planes: parasagittal (**left panels**), coronal (**middle panels**) and transverse (**right panels**). Dotted lines in the parasagittal view of (**A**) show planes of coronal (vertical line) and transverse (~horizontal line). (**B**) Three-dimensional reconstruction for the distal hindlimb, from which muscle volumes were determined. In all panels, blue is the left hindlimb, and green is the right hindlimb. (**C**,**E**) Quantitative data for distal (**C**) and proximal (**E**) muscle volumes. *p* values are the result of an unpaired *t*-test.

Magnetic Resonance Imaging (MRI) is commonly used to assess traumatic musculoskeletal abnormalities, and is potentially useful for longitudinal, non-invasive tracking of muscle atrophy reversal in CMT2A. To our knowledge, MRI has not been reported in a preclinical CMT2A model; we tested MRI in male rats whose large size made this technique feasible. Hindlimb muscles were imaged in three orthogonal views in male CMT2A rats and their male WT littermate controls (Figure 3A) and 3-dimensional reconstructions were generated (Figure 3B). Distal hindlimb muscle volumes of CMT2A rats averaged ~15% (*p* < 0.001) lower than those of their WT littermates (Figure 3C). As in the human condition, proximal hindlimb muscles in the same animals showed less severe involvement (~6% reduction vs. WT) that, nevertheless, approached statistical significance (Figure 3D,E).

### 2.3. Cellular Abnormalities in CMT2A Rats

**Figure 4:**
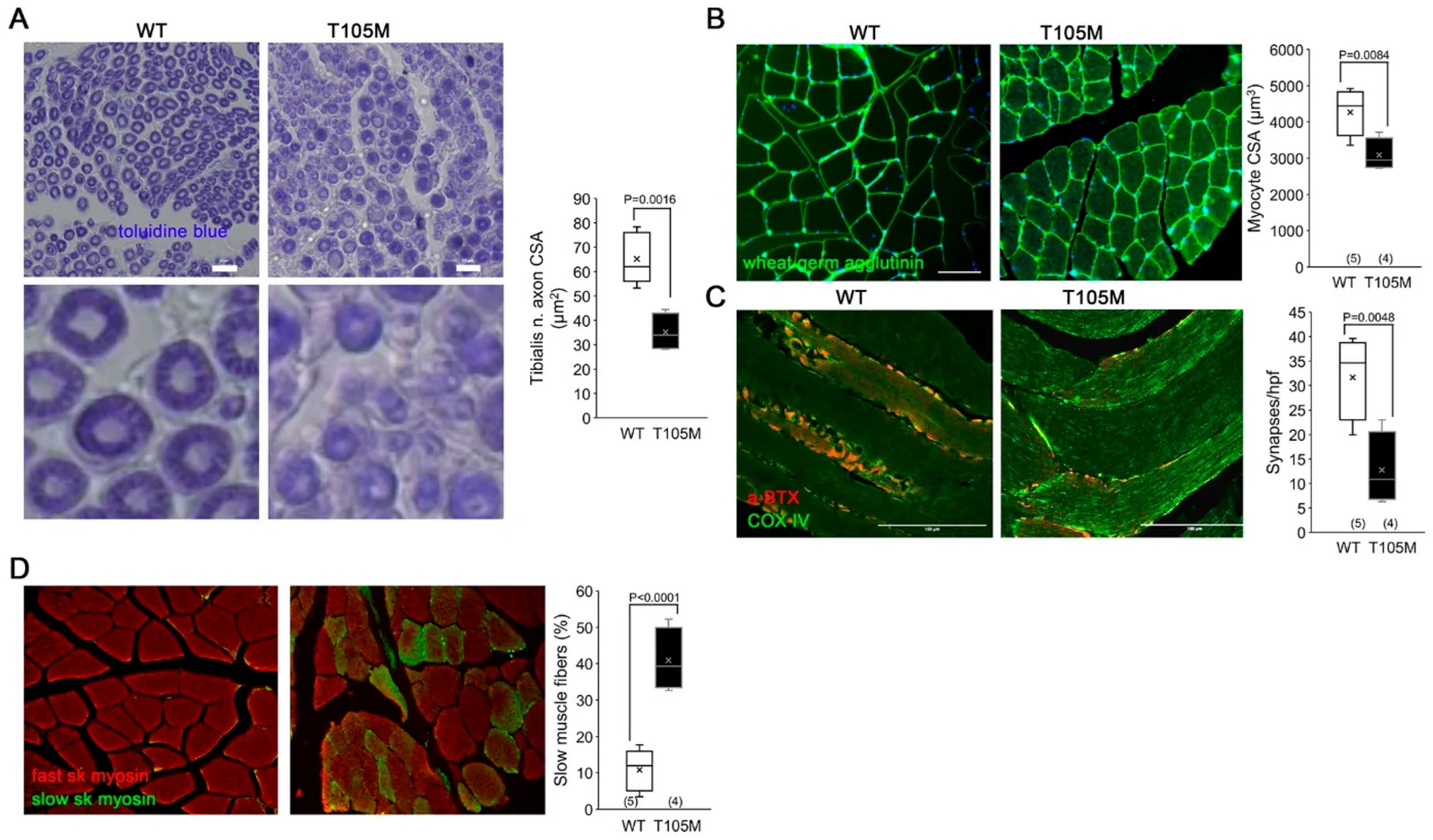
Histological findings in Mfn2 T105M KI rat hindlimbs. (**A**) Toluidine blue staining of tibialis nerve axons in cross section. CSA is cross sectional area. The scale bar (top panels) represents 20 microns; original magnification is 600×. (**B**–**D**) Gastrocnemius muscle: (**B**) Wheat germ agglutinin (green) staining of myocyte cell borders in cross section. The scale bar represents 20 microns. (**C**) α-bungarotoxin (BTX) staining (red) of neuromuscular synapses and mitochondrial cytochrome oxidase (COX) IV (green). The scale bar represents 100 microns. (**D**) Fast (red) and slow (green) skeletal (sk) myosin stain; same scale as (**C**). *p* values vs. WT are results of an unpaired 2-tailed *t*-test.

Human CMT2A is a non-demyelinating axonopathy. Toluidine blue staining of tibialis neurons from CMT2A rats revealed reduced average axonal diameters compared to those from normal rats (Figure 4A, top panels), but myelination was normal (Figure 4A, bottom panels).

Muscle histology in clinical CMT2A tends to show non-specific findings [23] such as myocyte atrophy, which was also observed in CMT2A rats (Figure 4B). Labeling of acetylcholine receptors in post-synaptic neuromuscular junctions with α-bungarotoxin revealed loss of neuromuscular synapses (Figure 4C), consistent with denervation.

The rat gastrocnemius is predominantly a fast-twitch muscle containing type 2 fibers that require an intact mitochondrial fission/fusion pathway [24] (Figure 4D, left panel). CMT2A rats showed a marked increase in hybrid muscle fibers expressing both fast and slow skeletal myosin (Figure 4D, right panel), which is typical of primary myopathy. Together, these data support both neuropathic and myopathic components of CMT2A.

### 2.4. Mitofusin Activator 8015-P2 Reverses Neuromuscular Dysfunction in CMT2A Rats

8015-P2 is a recently described third-generation small molecule mitofusin activator exhibiting high potency (EC_50_ < 1 nM) [15] and >10,000-fold specificity for mitofusin vs. a panel of 87 potentially cross-reacting drug targets (Supplemental Table S1). As 8015-P2 has properties that may favor clinical development, we tested its effects in CMT2A rats.

**Figure 5:**
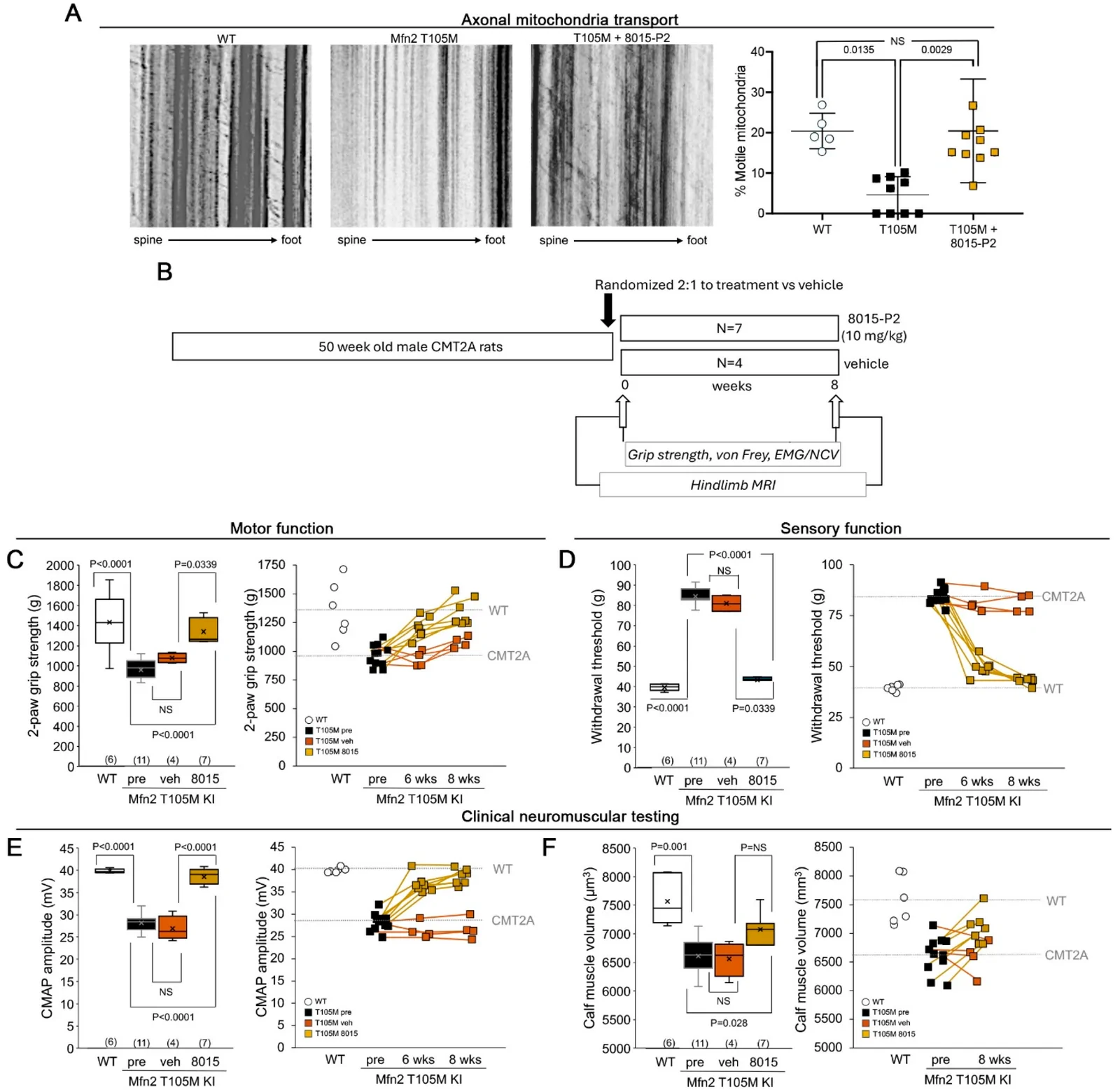
Reversal of neuromuscular phenotypes in Mfn2 T105M KI rats by mitofusin activator 8015-P2. (**A**) Mitochondrial motility studies. Shown are representative kymographs for WT (**left**), T105M (**middle**), and T105M 2 h after receiving 100 mg/kg 8015-P2 by oral gavage (**right**). Individual ex vivo neuron data are to the far right. (**B**) Schematic depiction of interventional trial design. (**C**–**F**) In vivo neuromuscular testing for grip strength (**C**), paw withdrawal (**D**), EMG/NCV CMAP amplitude (**E**), and MRI muscle volumes (**F**). Box and whisker graphs show group data after 8 weeks of treatment; scatter plots present individual rat results at 6 and 8 weeks (WT and CMT2A dashed lines are mean values for those animals). White is WT, black is pre-treatment T105M, orange is vehicle (veh)-treated T105M, and mustard is 8015-P2-treated T105M. *p* values used one-way ANOVA and Tukey’s test. NS = non-significant.

8015-P2 in vivo target engagement was assessed by measuring its effects on mitochondrial motility in neuronal axons of CMT2A rats. Defective mitochondrial transport provoked by loss of function MFN2 mutants is widely considered to be a major driver of neuropathy and denervation myopathy in CMT2A [25,26]. In support of this notion, only ~5% of total mitochondria were motile in CMT2A rat sciatic nerve axons compared with normal ~20% motile mitochondria in WT littermates (Figure 5A). However, two hours after oral administration of 8015-P2 (100 mg/kg), mitochondrial motility in CMT2A rat sciatic nerve axons was normalized (Figure 5A, mustard squares).

As systemic administration of 8015-P2 rapidly corrected a central aspect of axonal mitochondrial pathology thought to underlie neuromuscular degeneration, we measured neuromuscular readouts in a cohort of CMT2A rats randomized 2:1 to receive 8015-P2 (10 mg/kg by oral gavage, administered 5 days/week) or its vehicle (Figure 5B); functional phenotyping was performed prior to and 6 and 8 weeks after treatment. Both grip strength and paw sensitivity were normalized by 8015-P2 treatment in CMT2A rats, suggesting that 8015-P2 promoted motor and sensory nerve regrowth, whereas vehicle-treated rats showed no change from baseline (Figure 5C,D). Nerve regrowth was manifested as a marked reduction in the smallest quartile of neurons resulting in normalization of axon diameter distribution within tibialis nerves (Supplemental Figure S1) and EMG/NCV assessment of gastrocnemius/tibialis muscle CMAP amplitude, which also showed normalization in 8015-P2 treated animals (Figure 5E). Over the 8 week treatment period, distal hindlimb muscle volume measured by MRI significantly increased in 8015-P2 treated CMT2A rats, but not in vehicle-treated CMT2A controls (Figure 5F). Comparisons of individual animal responses revealed that 8015-P2 was consistently beneficial (Figure 5C–F, scatter plots).

### 2.5. Temporal Patterns of CMT2A Reversal After Mitofusin Activator Treatment

**Figure 6:**
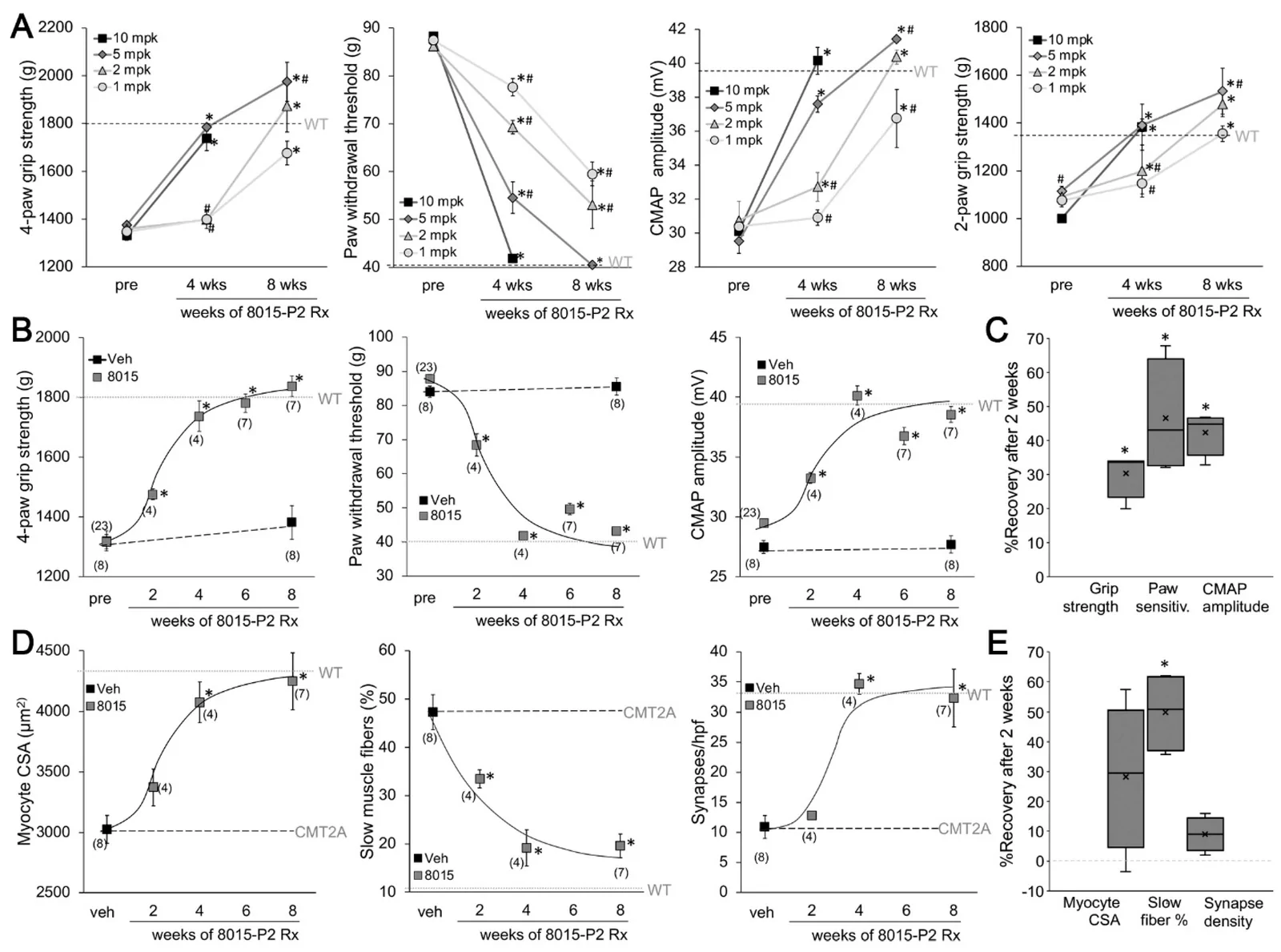
Dose- and time-dependency of CMT2A rat phenotype responses to mitofusin activation. (**A**) Dose–response studies (n = 4/group) for 4-paw grip strength (**left**), paw sensitivity (second), EMG/NCV CMAP amplitude (third), and front paw grip strength (**right**). Dashed lines indicate average WT response (n = 7). The 10 mg/kg (mpk) group was terminated after 4 weeks because it had reached normal values for all metrics. (**B**) Time course neuromuscular function study results in rats treated with 8015-P2 (10 mpk). (**C**) Relative recovery (% difference between baseline and peak response) at 2 weeks for studies in (B). (**D**) Muscle histopathology of rats in (**B**). (**E**) Relative recovery at 2 weeks for studies in (**D**). * = *p* < 0.05 vs. pre-treatment (**A**–**C**) or vehicle-treated rats (**D**,**E**); # = *p* < 0.05 vs. WT (one- or two-way ANOVA and Tukey’s test).

Nerve re-growth in mammals is limited to 1–2 mm/day [27], which would seem to limit the speed with which CMT2A-induced denervation myopathy can be restored by mitofusin activation. Alternatively, pathological suppression of mitofusin functioning by CMT2A-linked mutants such as MFN T105M might have direct myopathic effects beyond denervation, as suggested by mitochondrial abnormalities in CMT2A rat muscle. We considered that a more detailed interrogation of CMT2A phenotype reversal at different 8015-P2 doses and time points might provide additional insight. Accordingly, we randomized a new cohort of 50 week old male rats to a range of 8015-P2 doses (1, 2, 5, and 10 mg/kg orally 5 days/week) and performed functional evaluations after 4 and 8 weeks of treatment. The results of grip strength, paw sensitivity and EMG/NCV testing were qualitatively similar in that each 8015-P2 dose showed some benefit (i.e., *p* < 0.05 vs. pre-treatment; Figure 6A, points with asterisks). Notably, the 2 higher doses fully normalized function after 4 weeks (i.e., *p* = NS or better vs. WT; Figure 6A), whereas the 2 lower doses required longer treatment periods to achieve the same level of benefit. Thus, the 8015-P2 treatment time required for full recovery of neuromuscular dysfunction in CMT2A rats is inversely proportional to daily dose.

The observation that phenotype reversal was dose- and time-dependent prompted a follow-up study to better define the temporal pattern of phenotype reversal; effects of the most rapidly effective 8015-P2 dose (10 mg/kg orally, 5 days/week) were evaluated bi-weekly using both functional and histopathological endpoints. At this dose, CMT2A-linked neuromuscular dysfunction and muscle histopathology were fully normalized after 4 weeks of treatment; no further functional or histological improvement was seen at 6 or 8 weeks (Figure 6B,D). At the 2-week time point, however, functional metrics (strength, sensation, and EMG/NCV signal; Figure 6B,C) and histological slow muscle fiber reversal (Figure 6D,E [middle box]) had only partially improved (~30–50% vs. each animal’s baseline value), and there was little evidence of synaptic re-innervation (Figure 6D,E [right box]); the % increase in myocyte cross-sectional area vs. each rat’s pre-treatment value was more variable (CSA; Figure 6E, left box), which is a common finding in regenerating muscle [28].

## 3. Discussion

Our principal objective in these studies was to create a pre-clinical model of CMT2A that can be used to assess the efficacy of candidate therapeutics using tests that are standard in the human disease, but are difficult or not possible to perform in mice. Accordingly, we engineered into the rat genome the MFN2 T105M CMT2A variant, which in patients causes early onset and rapidly progressive disease [19]. Male and female heterozygous Mfn2 T105M rats exhibited readily measurable and highly consistent defects in strength and tactile responsiveness that, along with histological and ultrastructural findings, mimic the human condition. Importantly, the large size of our male rats enabled the evaluation of clinically relevant studies, such as MRI of muscle volume and EMG/NCV assessment of integrated neuromuscular function, to determine not just the efficacy of a potential therapeutic but also to establish quantitative in vivo dose–response and time–response relationships.

### 3.1. The Disease Model

CMT2A is associated with over 100 different mutations of MFN2 (https://www.hgmd.cf.ac.uk/ac/gene.php?gene=MFN2; accessed on 10 July 2026). However, many of these are reported in a single individual or family and most have not been biologically validated by interrogation of cellular effects or catalytic GTPase activity. The majority of CMT2A-linked MFN2 variants cluster in the MFN2 amino terminal GTPase domain and are presumed to adversely impact GTPase activity that is necessary for mitochondrial fusion [11]. Other pathologic variants cluster in the MFN2 hydrophobic core or carboxyl terminus [19]. Here, we chose to edit into the rat genome the GTPase domain mutation MFN2 T105 because it causes an early, severe, and aggressive form of human CMT2A [19]; its deleterious effects on mitochondrial function have been extensively characterized in CMT2A patient fibroblasts and motor neurons [13,15,29] and genetically modified mouse cells [14]; it lacks GTPase activity [20]; and it evokes strong phenotypes in mice when expressed either as a transgene [13,15,29] or as a recently described knock-in allele [15].

The genetics and neuromuscular phenotype of Mfn2 T105M knock-in rats strongly resemble those of Mfn2 T105M knock-in mice [15]. Mfn2 T105M homozygosity was embryonic lethal in both species, and although the tests employed differed in some instances, both heterozygous CMT2A models manifested diminished grasp strength and paw sensitivity, reduced EMG/NCV CMAP amplitudes with normal nerve conduction times, and microscopic skeletal muscle atrophy with evidence for denervation; neither species exhibited an alteration in tail thermal sensitivity. The major phenotypic difference between the Mfn2 T105M knock-in rat and mouse models is their performance on Rotarod testing. In mice, a reduced time to falling off the Rotarod device (latency) was a sensitive indicator of disease and therapeutic response to mitofusin activation [15]. By contrast, both wild-type control and CMT2A rats elected not to remain on the Rotarod and dismounted after a brief period, which we believe reflects species-specific behavioral differences rather than any disparity in neuromuscular function.

Comparing the phenotypes of Mfn2 T105M knock-in rats and Mfn2 T105M knock-in mice [15] may seem workmanlike, but is essential for interpreting differences in phenotypes like Rotarod performance. Because we edited into their respective genomes the same pathogenic *Mfn2* variant, phenotypic differences between CMT2A rats and mice can confidently be assigned to species differences rather than to possible effects of different CMT2A-linked MFN2 mutations. Moreover, the current studies break new ground by taking advantage of the size differences between mice and rats to expand the galaxy of CMT2A metrics and demonstrate their utility for following disease response to candidate therapies. The large size of male rats, approaching 800 g on average, enabled MRI muscle volume determinations that are not possible in mice. Larger size also provided greater confidence in results from other tests, wherein the small size of mice poses a challenge for available technology. Thus, EMG/NCV testing in mouse hindlimbs is limited by short mouse sciatic nerves (3–4 cm) and diminutive calf muscles, whereas male rat sciatic nerves are 10–11 cm in length and the larger calf muscles provided a more discriminative EMG signal.

Perhaps the most important translational aspect of this work is the detailed dose- and time-course studies with 8015-P2. The few published preclinical studies of potential therapeutics for CMT2A used a single drug dose with only one or two time points [12,13,14,15]. Our data establish both a minimum effective dose for 8015-P2 in CMT2A rats (2 mg/kg daily) that can be used to estimate dosage in future human trials and suggest that at least some disease metrics can show an early response to this form of therapy.

To our knowledge, only one other rat CMT2A model, an Mfn2 H361Y knock-in, has been reported in the scientific literature [30]. While there is a mention of “gait abnormalities” in these animals, the published manuscript does not include formal testing of neuromuscular function. Instead, the response to concomitant deletion of the *Sarm1* gene was described using microscopy. Such data are of interest, but the absence of functional testing seemingly limits clinical translatability. (Note—the PsychoGenics.com website refers to “two rat models of CMT2A expressing human mutations of MfN2” and presents minimal gait, grip strength and tail nerve conduction data for one line but does not identify the *Mfn2* gene variants: https://www.psychogenics.com/resources/characterization-of-a-rat-model-of-cmt2a/; accessed on 5 May 2026).

### 3.2. The Disease-Modifying Compound

Mitofusin activators are a recently described class of bioactive compounds that allosterically activate mitofusins 1 and 2. 8015-P2, the newest of this class, is the active *trans*-[(1R,2R)cyclopropyl] enantiomer of 8015, a third-generation reverse carboxamide mitofusin activator [15,31]. 8015-P2 represents the current evolution of previous-generation phenylhexanamide mitofusin activators such as MiM-111 and CPR1-B [13,14,32,33], which are modifications of prototype triazol-urea mitofusin activators such as Chimera [32,34]. Although their pharmaceutical properties differ, these small molecule mitofusin activators share essential structural features [15] that mimic biophysical properties of amino acid side chains in the MFN2 hydrophobic “zipper domain” (amino acids 367–384; [14]). Zipper domain amino acids interact with carboxyl terminal amino acids to maintain a folded/inactive mitofusin protein conformation [34,35]. In nature, non-phosphorylated mitofusins have a loosely coiled zipper domain α-helix in the hydrophobic core, resulting in weak peptide–peptide bonds that permit protein unfolding into the active conformation (“unzipped”). Mitofusin phosphorylation on Ser 378 tightens the zipper domain α-helix, enhancing peptide–peptide interactions and promoting the folded/inactive conformation [34,36]. By mimicking the zipper domain amino acid side chains, mitofusin activators can insert into and disrupt the interacting peptide pairs, thus evoking the unfolded mitochondrial conformation favoring mitochondrial fusion.

Mitofusin activation with first-generation Chimera corrected or improved mitochondrial abnormalities in cultured fibroblasts derived from patients with CMT2A, CMT1, Parkinson’s disease and amyotrophic lateral sclerosis [29,37], suggesting broad utility for this class of compounds in neurodegenerative diseases having a mitochondrial component [38]. However, first-generation triazol-urea mitofusin activators like Chimera proved to be pharmaceutically unacceptable for in vivo use because of first pass metabolism [32]. Second-generation MiM-111 and CPR1-B were active in vivo and have been used as proof-of-principle that mitofusin activation can ameliorate mitochondrial damage and neuromuscular dysfunction in mouse models of CMT2A and amyotrophic lateral sclerosis [13,14,37]. Although 8015-P2 has not yet been tested in models other than CMT2A, the collective results of mitofusin activator studies support the notion that enhancing mitofusin-mediated mitochondrial fusion can correct mitochondrial damage of diverse etiology [39].

Like previous generations of mitofusin activators, 8015-P2 interacts directly with mitofusin proteins and exhibits stereoselective fusogenicity, indicating that its biological activity is mediated via the mitofusin interactions [15]. Indeed, 8015-P2 exhibits the same fusogenic activity with Mfn1 or Mfn2, but has no fusogenic effects in cells lacking both mitofusin proteins [15]. Moreover, the EC_50_ for 8015-P2 to stimulate mitochondrial fusion is ~0.6 nM [15], whereas a survey of 8015-P2 (10 μM) interactions with 87 G-protein coupled receptors, ion channels, enzymes, transporters, and nuclear receptors found that the only off-target interaction affecting activity or binding by at least 50% was for monoamine oxidase-A (59% inhibition with 10 μM 8015-P2) (see Supplemental Table S1). Thus, 8015-P2 exhibits greater than 10,000-fold specificity for mitofusins, making it a useful addition to the research armamentarium.

### 3.3. The Disease Markers

Evaluating CMT2A in human patients is challenging because of the rarity (1:60,000) and genetic heterogeneity of the disease [19]. Indeed, determining the clinical status of CMT2A patients at initial presentation, and phenotypes of CMT2A mouse models, has relied almost entirely upon functional scoring and EMG/NCV testing [11,13,14,15,17,40]. We recognized the need to generate a pre-clinical model in which disease metrics can be assessed not only for their ability to discriminate CMT2A from normal (i.e., WT), but also as surrogate endpoints to gauge CMT2A response to candidate therapeutics in future clinical trials [41]. The current results support the examination of skeletal muscle to provide information that can augment results of standard neurological testing. Histological assessment of myocyte cross sectional area combined with immunohistological determination of neuromuscular synapse density and fast/slow myofiber content not only identified CMT2A, but also reflected or anticipated functional improvement after pharmacological mitofusin activation. Because thin needle muscle biopsy is commonly used to diagnose clinical neuromuscular disorders [42], it would be feasible to obtain these types of data in serial fashion from CMT2A patients. Quantifying limb muscle volume using MRI provided another independent metric of CMT2A response that can be readily translated to the clinic.

### 3.4. Insights Relating to CMT2A

It might seem remarkable that sensory-motor dysfunction and muscle histopathology in CMT2A rats could be fully normalized after only four weeks of high-dose 8015-P2 treatment. However, this result is consistent with previous studies wherein mitofusin activators MiM-111, CPR1-B or 8015-P2 corrected neuromuscular phenotypes in various CMT2A mouse models after weeks of treatment [13,14,15]. Moreover, the current results are internally consistent across multiple independent endpoints and demonstrate dose–response and time-course relations that support a rapid therapeutic effect of mitofusin activation in this model. Accordingly, we conclude by considering the possible implications of rapid disease reversal:

Conventional wisdom holds that truncated nerves can regenerate only 1–2 mm/day [27]. In this context, how is it that neuromuscular dysfunction in CMT2A rats is fully corrected within a few weeks of treatment? Correction of a primary myopathy component of CMT2A separate from secondary denervation myopathy is not dependent upon neuronal regrowth and could accelerate or augment functional and histological recovery. Indeed, muscle repair and regrowth can occur quite rapidly [43]. Moreover, the exact nature of the “nerve die-back” phenomenon in CMT2A has not been established. Neuronal pathology in CMT2A may not simply be physical dying back of neurons causing premature nerve termination at the axon, but may include pruning of distal synapses that can more rapidly be reversed than axonal regeneration through synaptic regrowth and collateral sprouting. In any case, the rapidity of response in CMT2A rats and mice is a positive prognostic indicator for possible disease reversal by mitofusin activation in CMT2A patients.

## 4. Methods and Materials

### 4.1. Sex as a Biological Variable

Our study examined both male and female animals. The data are reported separately for male and female rats because of differences in size and strength. Only male rats were used in disease reversal studies because their larger size was essential for some of the analytical techniques employed.

### 4.2. Mfn2 T105M Mutant Rat Line

All procedures involving live animals were approved by the Medical University of South Carolina (MUSC) Institutional Animal Care & Use Committee, protocol number AUP-25-22 (16 May 2025). Rats were maintained on a 12 h light/dark cycle with ad libitum access to food and water.

CRISPR/Cas9 replacement of rMfn2 T105 (ACG codon) with M105 (ATG codon) was performed at Cyagen Inc., as schematically depicted, including gRNA and ssODN sequences in Figure 1A. The ACG to ATG mutation in the donor oligo was introduced into rMfn2 exon 4 by homology-directed repair: Super-ovulated female Sprague–Dawley (SD) rats were mated to SD males to obtain single cell fertilized embryos for pronuclear injection and transfer to pseudo-pregnant foster mothers. Rat pups (~7 days old) were genotyped by Sanger sequencing to confirm correct integration of the single nucleotide mutation. Founders were back-crossed to SD breeders to obtain heterozygous F1s. Mutant F1 breeders were inter-bred to obtain experimental animals, all of which were either heterozygous Mfn2 T105M or wild-type Mfn2 T105 (WT; ~2:1 ratio). All breeder and experimental animals were genotyped by Sanger sequencing. Mfn2 T105M rats used in the current study were F2-F4.

### 4.3. Functional Studies

Based on the published observations that neuromuscular phenotypes develop progressively over approximately 1 year in motor neuron-specific Mfn2 T105M transgenic mice [13,14] and Mfn2 T105M knock-in mice [15], we maintained heterozygous rMfn2 T105M rats and their WT littermate controls for 50 weeks prior to detailed phenotyping. Functional testing was performed in the mornings in a separate laboratory after acclimatization for a minimum of 30 min.

### 4.4. Sensory Neuron Function

*Thermal sensation* was measured by tail immersion in hot water. Rats were individually restrained in a plexiglass unit (Zucker Laboratory rat restrainer, 500–800 g) with the tail exposed. After acclimatization for at least 2 min, the distal 5 cm of the tail was immersed in 52° water. The time until tail flicking or withdrawal was measured. Each daily study was performed 3 times and the average withdrawal latency reported.

*Mechanical sensation* was measured as the force needed to provoke foot pad withdrawal in response to prodding with a rigid nylon filament (von Frey test). Rats were individually placed in an isolation chamber with an open grid floor and acclimated for at least 5 min. An electronic von Frey anesthesiometer (IITC Life Science, Woodland Hills, CA, USA) with a rigid tip was used to gradually apply force to the hind paw until it was withdrawn, and the maximum force applied was recorded. The test was then repeated on the opposite hind paw; 10 independent measurements (5 per paw) were recorded per animal on each study day and the average withdrawal threshold force (in grams) reported.

### 4.5. Motor Neuron Function

*Grip strength* was measured as the maximal force required to pull a rat off a horizontal metal grid when gripped with either the two front paws only, or with all four paws. Rats were placed on the grid of a Bioseb BIO-GS3 instrument (Vitrolles, France) and allowed to grip either with just the front paws (the rear of the body being supported by the investigator) or all 4 paws. The rat was pulled horizontally until it released, and the maximal tension (force in grams) was recorded. The maximal withdrawal force in 8 independent measurements (4/day over 2 consecutive trial days) was reported.

*Rotarod testing* was performed on an IITC Roto-Rod Series 8 instrument with a 3.75 inch diameter rotor (IITC Inc./Life Science, Woodland Hills, CA, USA). The Rotarod was accelerated from 0 to 10 RPM over a period of 120 s, and then maintained at that speed for a maximum of 180 s. Both WT and CMT2A rats electively dismounted approximately 60 s into the test, which was not discriminative.

### 4.6. Neuroelectrophysiology

Electromyography/nerve conduction velocity (EMG/NCV) testing was performed by stimulating the proximal sciatic nerve and measuring depolarization/repolarization of the combined rear hindlimb calf muscles (gastrocnemius/soleus/tibialis) with a Nicolet VikingQuest EMG system (MFI Medical, San Diego, CA, USA) running version 11.2 software. The rat EMG/NCV procedure was adapted from that previously described for mice [13,14,15]. Briefly, rats were anesthetized using 3–3.5% isoflurane with 1.5 L/min supplemental oxygen flow and maintained on a heating pad. The para-lumbar regions and hindlimb calf were shaved and the study leg extended and fixed with adhesive tape. A ground needle electrode was placed under the skin at the posterior neck, two stimulating electrodes inserted subcutaneously 2–3 cm apart at the lumbar spine along the track of the sciatic nerve, and two loop electrodes placed at the maximum diameter of the gastrocnemius muscle (sensing electrode) and just above the ankle (reference electrode). Stimuli were programmed as single 25 mA pulses of 0.1 msec duration; recording scale was initially set at 10 mV. Electrodes were repositioned as necessary to optimize CMAP configuration and amplitude. A minimum of three separate recordings were obtained per animal per study session, and the animal permitted to recover on a heating pad. CMAP amplitude is measured from baseline to peak; nerve conduction time is the interval between stimulus spike and initial deflection. The three best action potential recordings were selected for analysis; reported CMAP amplitude and nerve conduction time values are the average of these three.

### 4.7. Magnetic Resonance Imaging

MRI quantification of hindlimb muscle volumes used a 9.4T MRI instrument (Bruker Biospec, Ettlingen, Germany) controlled by ParaVision 360 3.5 using an 86 mm MRI transceiver coil. Rats were anesthetized with isoflurane in oxygen adjusted for respiration frequency (4% for induction, 2% for maintenance). Animal temperatures were maintained using a circulating warm water pad. Rats were positioned supine with their legs secured in flexion using a custom 3D-printed support. Trans-axial DIXON fat–water-separated T2-weighted RARE images of the femurs and tibias were acquired separately (Rare Factor = 6, Effective TE = 22.4 ms, TR = 4053 ms, 0.4 × 0.4 mm resolution, 70 slices of 0.8 mm thickness, number of averages = 2). The total scanning time per animal was 35 min. Muscle volumes of the right and left lower and upper legs were calculated using ITK-SNAP software (version 4.2.2). Briefly, muscles were manually outlined using the segmentation tool, initially in the transverse plane and subsequently in the parasagittal and coronal planes, and the average of right and left muscle volume reported for each animal.

### 4.8. Mitochondrial Transport in Rat Sciatic Nerve Axons

Mitochondrial motility was measured using a modification of the technique previously reported for mice [13,15]. Briefly, the lumbar spine and sciatic nerve extending to the tibialis bifurcation (~knee level) were removed en bloc, dissected clean, and maintained in 37 °C Gibco Neurobasal medium lacking phenol red and containing 200 nM tetramethyl rhodamine ester (TMRE; Invitrogen cat# T669, Thermo Fisher Scientific, Waltham, MA, USA) for 30 min. After rinsing, the nerve underwent time-lapse video imaging on a Nikon Eclipse Ti2 confocal microscope (1 frame/5 s for 15 min). Kymographs of mitochondrial movement were generated from time-lapse image files using the Image J (version 1.54p) Kymograph plug-in.

### 4.9. Tissue Histology, Immunostaining and Enzyme Activity Stains

Rat gastrocnemius muscles, and sciatic and tibial nerves, were removed immediately after sacrifice (anesthesia overdose followed by terminal thoracotomy) and prepared for frozen sections, or fixed in 4% paraformaldehyde (PFA) or 2.5% glutaraldehyde as required.

*Myocyte cross sectional area* (CSA), *neuromuscular synapse density*, and the ratio of *fast/slow myofibers* were measured in muscle samples initially fixed for ~2 h in 4% PFA, cryoprotected in 30% sucrose overnight, and then frozen in optimal cutting temperature (OCT) medium using liquid nitrogen-cooled isopentane. Embedded samples were stored at −80° C until cryostat sectioning (10 μm thickness). Myocyte CSA and fast/slow myofiber staining was performed on sections showing myocytes in cross-section; synapse density was assayed in sections showing myocytes in the longitudinal orientation. Sections were fixed in −20° acetone followed by antigen retrieval in boiling citrate buffer and blocking in 10% goat serum in phosphate-buffered saline (PBS). Sections were stained using standard techniques and the following reagents: *CSA*—Alexa Fluor 350-tagged wheat germ agglutinin (WGA; Invitrogen cat# W11263, final concentration 0.02 mg/mL); *fast/slow myosin*—rabbit monoclonal anti-slow myosin heavy chain (MYH7; Abcam (Cambridge, UK), cat# ab234431, 1:200), mouse monoclonal anti-fast myosin skeletal heavy chain (MYH1 and MYH2; Abcam, cat# ab51263, 1:750); *Neuromuscular synapses*—Alexa Fluor 594-labeled α-bungarotoxin (Invitrogen cat# B13421, 1:200), and rabbit polyclonal anti-cytochrome c oxidase (COX IV; Abcam cat# ab16056, 1:1000). Secondary antibodies were Alexa Fluor 488-labeled goat anti-rabbit antibody (Invitrogen, cat# A11008) and Alexa Fluor 594-labeled goat anti-mouse antibody (Invitrogen, cat# A32742). Cover slips were mounted using Fluoroshield (Abcam cat# ab104135). Images were acquired on a Nikon Eclipse Ti2 confocal microscope using a 20× dry objective (WGA and fast/slow myofibers) or a 60× water immersion objective (neuromuscular synapse density). Image J version 1.54p was used to quantify myocyte CSA.

### 4.10. Mitofusin Activator Treatment

Mitofusin activator 8015-P2 [15] (>99% pure in powder form) was a gift from Mitochondria in Motion Discovery, Inc. (Sullivan’s Island, SC, USA). 8015-P2 was prepared fresh weekly. Briefly, a stock solution was made by dissolving 250 mg of drug in 1 mL dimethyl sulfoxide (DMSO) with vortexing and heating at 56 °C for 30 min. Working solutions (typically 10 mg/mL) were prepared from the stock by adding 8015-P2 in DMSO to 30% hydroxypropyl-β-cyclodextrin (HP-β-CD; Roquette Kleptose, Lestrem, France) with vortexing and heating to 56 °C for 30 min or until clear. Stock and working solutions were stored at 4 °C; working solutions were brought to room temperature before use.

After baseline phenotyping at 50 weeks of age, CMT2A rats were randomized to treatment with 8015-P2 or vehicle using the random number generator in Excel. Drug or vehicle (30% HP-β-CD) were administered daily every Monday–Friday in the mornings via oral gavage using a 3-inch-long ball-tipped 18 g straight gavage needle coupled to a 1 mL syringe. Study duration ranged from 2 to 8 weeks, and 8015-P2 dose ranged from 1 to 10 mg/kg/day as specified for individual results. All in vivo functional testing and postmortem pathological analyses were performed in a blinded manner.

### 4.11. Statistical Analyses

Unless otherwise stated, results are presented as bar and whisker plots wherein the whiskers show the range of values, the bar encompasses the 1st–3rd interquartile range, the internal horizontal line is the median value, and the internal X is the mean value. Line graphs report means ± SEM. Paired comparisons used the unpaired Student’s *t*-test; multi-group comparisons used one- or two-way ANOVA as specified, with Tukey’s post hoc for comparison. *p* < 0.05 was considered significant.

## 5. Conclusions

The evidence we present suggests direct muscle involvement in CMT2A and supports the need to investigate phenotypes beyond the nervous system, i.e., in other organs that are highly dependent upon mitochondria; the Mfn2 T105M rat may prove useful for this purpose. A paradigm of systemic mitochondrial dysfunction in CMT2A argues against therapeutic efforts that exclusively target the nervous system in this condition.

## Data Availability

Primary data are available in the Data Supplement.

## Supplementary Materials

The following supporting information can be downloaded at: https://www.mdpi.com/article/10.3390/ijms27167376/s1.
