# Supplementary material for: Neuromuscular Dysfunction and Charcot-Marie-Tooth Disease Reversal in Mfn2 T105M Knock-In Rats
Source: Int J Mol Sci. 2026 Aug 18;27(16):7376. doi: 10.3390/ijms27167376 (PMC13513834; doi:10.3390/ijms27167376)
Supplement: Supplementary file 1 [file ijms-27-07376-s001.zip › Supplemetnal Table S1.pdf]

Supplemental Table S1. *Results of Eurofin SafetyScreen 87 Panel for off-target interactions of 8015-P2.* All studies used 10 µM 8015-P2, i.e. ~10,000 times its EC_{50} of ~0.8 nM. The only interaction >50% at this concentration is monoamine oxidase (59%).

==			
**Binding assays**			
==			
**Assay**	**Compound**	**% Inhib**	**Reference Cpd**
Adenosine Guinea Pig Transporter Mass Spectrometry Binding (Antagonist Ligand) Assay, Cerep	8015-P2	26	Dilazep
Non-Selective Rat Glycine Ion Channel Strychnine Mass Spectrometry Binding Assay, Cerep	8015-P2	-1	guanabenz
Glutamate (Non-Selective) Rat Ion Channel Glycine (Strychnine-Insensitive),Mass Spectrometry Binding Assay, Cerep	8015-P2	-2	5,7 dichlorokynurenic acid
GABAB(B1b/B2) Human GABA B GPCR Mass Spectrometry Binding (Antagonist Ligand) Assay, Cerep	8015-P2	15	CGP 52432
A1 (h) (antagonist radioligand)	8015-P2	-16	DPCPX
A2A (h) (agonist radioligand)	8015-P2	-2	NECA
alpha1A (h) (antagonist radioligand)	8015-P2	35	WB 4101
alpha1B (h) (antagonist radioligand)	8015-P2	33	prazosin
alpha1D (h) (antagonist radioligand)	8015-P2	43	prazosin
alpha2A (h) (antagonist radioligand)	8015-P2	6	yohimbine
alpha2B (h) (antagonist radioligand)	8015-P2	17	yohimbine
beta1 (h) (agonist radioligand)	8015-P2	1	atenolol
beta2 (h) (antagonist radioligand)	8015-P2	-3	ICI 118551
AT1 (h) (antagonist radioligand)	8015-P2	8	saralasin
BZD (central) (agonist radioligand)	8015-P2	-4	diazepam
Cl- channel (GABA-gated) (TBOB site) (antagonist radioligand)	8015-P2	-3	Picrotoxin
B2 (h) (agonist radioligand)	8015-P2	-4	NPC 567
CB2 (h) (agonist radioligand)	8015-P2	33	WIN 55212-2
CB1 (h) (agonist radioligand)	8015-P2	-36	CP 55940
CCK1 (CCKA) (h) (agonist radioligand)	8015-P2	9	CCK-8s
CCK2 (CCKB) (h) (agonist radioligand)	8015-P2	2	CCK-8s
D1 (h) (antagonist radioligand)	8015-P2	5	SCH 23390
D2S (h) (agonist radioligand)	8015-P2	19	7-OH-DPAT
D2L (h) (antagonist radioligand)	8015-P2	-1	butaclamol
ETA (h) (agonist radioligand)	8015-P2	2	endothelin-1
GABAA1 (h) (alpha1,beta2,gamma2) (agonist radioligand)	8015-P2	4	muscimol
AMPA (agonist radioligand)	8015-P2	-5	L-glutamate
kainate (agonist radioligand)	8015-P2	-9	kainic acid
NMDA (antagonist radioligand)	8015-P2	-2	CGS 19755

mGluR5 (h) (agonist radioligand)	8015-P2	-6	L-Quisqualate
CXCR2 (IL-8B) (h) (agonist radioligand)	8015-P2	-4	IL-8
CCR1 (h) (agonist radioligand)	8015-P2	-2	MIP-1alpha
H1 (h) (antagonist radioligand)	8015-P2	-6	pyrilamine
H2 (h) (antagonist radioligand)	8015-P2	-9	cimetidine
CysLT1 (LTD4) (h) (agonist radioligand)	8015-P2	-14	LTD4
MC1 (agonist radioligand)	8015-P2	6	NDP-alpha -MSH
MC4 (h) (agonist radioligand)	8015-P2	-1	NDP-alpha -MSH
MAO-A (antagonist radioligand)	8015-P2	59	clorgyline
M1 (h) (antagonist radioligand)	8015-P2	1	pirenzepine
M2 (h) (antagonist radioligand)	8015-P2	-4	methoctramine
M3 (h) (antagonist radioligand)	8015-P2	-5	4-DAMP
M4 (h) (antagonist radioligand)	8015-P2	2	4-DAMP
NK1 (h) (agonist radioligand)	8015-P2	12	[Sar9,Met(O2)11]-SP
Y1 (h) (agonist radioligand)	8015-P2	-4	NPY
N neuronal alpha4beta2 (h) (agonist radioligand)	8015-P2	3	nicotine
N muscle-type (h) (antagonist radioligand)	8015-P2	-15	alpha -bungarotoxin
delta (DOP) (h) (agonist radioligand)	8015-P2	1	DPDPE
kappa (h) (KOP) (agonist radioligand)	8015-P2	8	U50488
mu (MOP) (h) (agonist radioligand)	8015-P2	21	DAMGO
PPARgamma (h) (agonist radioligand)	8015-P2	7	rosiglitazone
PCP (antagonist radioligand)	8015-P2	-2	MK 801
PAF (h) (agonist radioligand)	8015-P2	0	C16-PAF
RARalpha (h) (agonist radioligand)	8015-P2	-7	all-trans-retinoic acid
5-HT1A (h) (agonist radioligand)	8015-P2	40	8-OH-DPAT
5-HT1B (h) (antagonist radioligand)	8015-P2	-1	Serotonine
5-HT2A (h) (agonist radioligand)	8015-P2	30	(±)DOI
5-HT2B (h) (agonist radioligand)	8015-P2	42	(±)DOI
5-HT2C (h) (antagonist radioligand)	8015-P2	7	RS 102221
5-HT3 (h) (antagonist radioligand)	8015-P2	2	MDL 72222
GR (h) (agonist radioligand)	8015-P2	2	dexamethasone
ER Alpha Human Estrogen NHR Binding (Agonist radioligand) Assay, Cerep	8015-P2	-3	Diethylstilbestrol
PR (h) (agonist radioligand)	8015-P2	5	promegestone
AR(h) (agonist radioligand)	8015-P2	10	testosterone
V1a (h) (agonist radioligand)	8015-P2	-1	[d(CH2)51,Tyr(Me)2]-AVP
Ca2+ channel (L, dihydropyridine site) (antagonist radioligand)	8015-P2	10	nitrendipine
Ca2+ channel (L, diltiazem site) (benzothiazepines) (antagonist radioligand)	8015-P2	-15	diltiazem
Ca2+ channel (L, verapamil site) (phenylalkylamine) (antagonist radioligand)	8015-P2	-10	D 600
Ca2+ channel (N) (antagonist radioligand)	8015-P2	0	omega -conotoxin GVIA

Potassium Channel hERG (human)- [3H] Dofetilide	8015-P2	-14	Terfenadine
KV channel (antagonist radioligand)	8015-P2	3	alpha -dendrotoxin
Na+ channel (site 2) (antagonist radioligand)	8015-P2	-13	veratridine
norepinephrine transporter (h) (antagonist radioligand)	8015-P2	-3	protriptyline
dopamine transporter (h) (antagonist radioligand)	8015-P2	28	BTCP
GABA transporter (antagonist radioligand)	8015-P2	5	nipecotic acid
5-HT transporter (h) (antagonist radioligand)	8015-P2	4	imipramine
**==**			
**Enzyme and Cell-based assays**			
**==**			
**Assay**	**Compound**	**% Inhib**	**Reference Cpd**
COX1(h)	8015-P2	6	Diclofenac
COX2(h)	8015-P2	5	NS398
PDE3A (h)	8015-P2	2	milrinone
PDE4D2 (h)	8015-P2	-12	Ro 20-1724
ACE (h)	8015-P2	-4	captopril
cathepsin G (h)	8015-P2	-28	3',4' dichloroisocoumarin
IRK (h) (InsR)	8015-P2	3	staurosporine
Lck kinase (h)	8015-P2	5	staurosporine
PKCalpha (h)	8015-P2	-2	Bis 10
acetylcholinesterase (h)	8015-P2	-3	galanthamine
MAO-B (h) recombinant enzyme	8015-P2	-5	deprenyl
ATPase (Na+/K+)	8015-P2	4	ouabain
